# Proximity to Crops and Residential Exposure to Agricultural Herbicides in Iowa

**DOI:** 10.1289/ehp.8770

**Published:** 2006-02-02

**Authors:** Mary H. Ward, Jay Lubin, James Giglierano, Joanne S. Colt, Calvin Wolter, Nural Bekiroglu, David Camann, Patricia Hartge, John R. Nuckols

**Affiliations:** 1 Division of Cancer Epidemiology and Genetics, National Cancer Institute, National Institutes of Health, Department of Health and Human Services, Bethesda, Maryland, USA; 2 Iowa Geological Survey, Iowa City, Iowa, USA; 3 Department of Biostatistics, Marmara University Medical School, Istanbul, Turkey; 4 Southwest Research Institute, San Antonio, Texas, USA; 5 Department of Environmental and Radiological Health Sciences, Colorado State University, Fort Collins, Colorado, USA

**Keywords:** agriculture, exposure assessment, geographic information systems, herbicides, pesticides

## Abstract

Rural residents can be exposed to agricultural pesticides through the proximity of their homes to crop fields. Previously, we developed a method to create historical crop maps using a geographic information system. The aim of the present study was to determine whether crop maps are useful for predicting levels of crop herbicides in carpet dust samples from residences. From homes of participants in a case–control study of non-Hodgkin lymphoma in Iowa (1998–2000), we collected vacuum cleaner dust and measured 14 herbicides with high use on corn and soybeans in Iowa. Of 112 homes, 58% of residences had crops within 500 m of their home, an intermediate distance for primary drift from aerial and ground applications. Detection rates for herbicides ranged from 0% for metribuzin and cyanazine to 95% for 2,4-dichlorophenoxyacetic acid. Six herbicides used almost exclusively in agriculture were detected in 28% of homes. Detections and concentrations were highest in homes with an active farmer. Increasing acreage of corn and soybean fields within 750 m of homes was associated with significantly elevated odds of detecting agricultural herbicides compared with homes with no crops within 750 m (adjusted odds ratio per 10 acres = 1.06; 95% confidence interval, 1.02–1.11). Herbicide concentrations also increased significantly with increasing acreage within 750 m. We evaluated the distance of crop fields from the home at < 100, 101–250, 251–500, and 501–750 m. Including the crop buffer distance parameters in the model did not significantly improve the fit compared with a model with total acres within 750 m. Our results indicate that crop maps may be a useful method for estimating levels of herbicides in homes from nearby crop fields.

People living in agricultural areas may be exposed to pesticides through drift from agricultural fields in proximity to their homes. In orchard-producing areas of Washington State, pesticide levels in carpet dust and pesticide metabolites in urine of residents increased with self-reported proximity of homes to crop fields (Lu et al. 2000) and during the pesticide application season (Curl et al. 2002). Children in agricultural areas had five times the concentration of pesticides in their urine compared with children in an urban area (Lu et al. 2000). The presence of an agricultural worker in the home also increases pesticide levels through “take-home” exposures (Curl et al. 2002; Curwin et al. 2005; Lu et al. 2000).

Carpet dust can be a reservoir for pesticides and other chemicals because they are protected from degradation. Levels of pesticide in carpet dust were 10- to 200-fold higher than levels in soil around the home in residential (Lewis et al. 1994) and agricultural areas (Simcox et al. 1995). Previous studies have found residues in carpet dusts from both recently used pesticides (Colt et al. 1998; Curwin et al. 2005; Lu et al. 2000) and pesticides used many decades ago (Colt et al. 1998, 2004; Rudel et al. 2003).

We previously developed a geographic information system (GIS)-based method that used satellite imagery to create historical crop maps in the midwestern United States. Residences were mapped, and the extent of agricultural fields proximate to the homes was determined as a way of identifying homes with potential exposure to agricultural pesticides (Ward et al. 2000). Corn and soybeans are the major crops in Iowa; > 95% of corn and soybean acreage was treated with herbicides from the late 1970s through the 1990s (Iowa State University 1997). The aim of the present study was to determine whether crop maps are useful for predicting residential levels of crop herbicides as determined by their measurement in carpet dust samples from residences in a population-based case–control study of non-Hodgkin lymphoma (NHL).

## Materials and Methods

### Study population and data collection

The study population in these analyses included Iowa case and control participants for whom we collected a dust sample in a population-based case–control study of NHL. Cases were included in these analyses because proximity to crops and herbicide concentrations in dust were not associated with risk of NHL. The original study was conducted in four areas covered by the Surveillance, Epidemiology, and End Results program of the National Cancer Institute (Iowa; the Detroit, Michigan, metropolitan area; Los Angeles County, California; and the Seattle, Washington, metropolitan area), and the study design was described in detail previously (Chatterjee et al. 2004; Colt et al. 2004). Briefly, we identified cases newly diagnosed with NHL between July 1998 and March 2000 and 20–74 years of age. We selected controls 20–64 years of age from the general population of Iowa using random-digit dialing and controls 65–74 years of age from Medicare eligibility files. Controls were matched by age group, sex, and race to cases. We interviewed 361 (67%) of eligible cases in Iowa and 276 (58%) of eligible controls.

**Figure 1:**
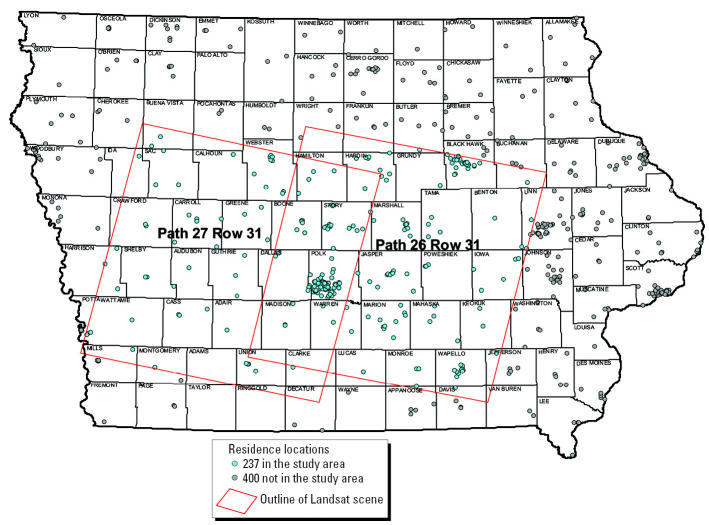
Satellite image boundaries and approximate residence locations from a case–control study of NHL in Iowa, USA.

We collected vacuum cleaner dust if participants had used their vacuum in the past year and had owned half of their carpets for ≥5 years. A further criterion for inclusion in these analyses was that a 750-m radius buffer around the home was within the area of the two satellite images (128 of 237 participants had dust samples and resided in the image area) (Figure 1). Using a GIS and spring and summer Landsat multispectral satellite images (U.S. Geological Survey Earth Resources Observation and Science 2006) from two path/rows in south central Iowa (hereafter called study area), we created land cover maps to identify corn and soybean fields (> 90% of crops) and other land uses for each year of the study (1998–2000), and we used Farm Service Agency records for validation (Ward et al. 2000). For the three maps, the accuracy (percentage of sampled areas on the crop maps for which the land cover was correctly identified) ranged from 81 to 96% for corn and from 87 to 93% for soybeans.

We determined the location of homes by global positioning system (GPS) measurements using the Garmin GPS12 Personal Navigator, a 12-channel small handheld receiver (Garmin International Inc., Olathe, KS, USA). Interviewers took the measurements 6.1 m (20 feet) away from the home. Quality control checking of the GPS locations was done by comparing addresses with GPS locations and using digital aerial photography, rural directories, and other data as previously described (Ward et al. 2005). One participant was excluded because of an inaccurate address. GPS locations that were not verified were remeasured by taking another GPS measurement at the street in front of the home. The estimated accuracy of the GPS measurements is 20 m (Ward et al. 2005). As previously described (Ward et al. 2005), residences were classified as “in town” if they were located inside of incorporated areas, and as rural otherwise.

### Laboratory analyses

Details of the procedures for shipping samples, sieving, batching, and quality control of the laboratory analyses have been described (Colt et al. 2004). We measured the concentrations of 14 agricultural herbicides, each of which was used on at least 15% of corn and/or soybean acres in Iowa in 1985, 1990, or 1995 (Iowa State University 1997). These analytes included 10 neutral-extractable herbicides (acetochlor, alachlor, atrazine, cyanazine, fenoxyprop ethyl, fluazifop-*p*-butyl, metolachlor, metribuzin, pendimethalin, trifluralin) and four acid-extractable herbicides [2,4-dichlorophenoxyacetic acid (2,4-D), bromoxynil, bentazon, dicamba]. Some corn and soybean herbicides that were used this extensively were not included because they could not be measured using gas chromatography/mass spectrometry (GC/MS) (thifensulfuron, nicosulfuron, chlorimuron) or because they could not be extracted using the neutral- or acid-extraction methods (imazethapyr, glyphosate).

We analyzed neutral and acid extracts using GC/MS in selective ion mode. Analyte amounts were quantified using the internal standard method. For the neutral extractions, 1 or 2 g of sieved fine dust was spiked with atrazine-d_5_ and isopropalin as surrogates. The spiked dust samples were sonicated and extracted with ether:hexanes (1:1), and the extracts were cleaned through a Florisil column. The acid extractions were performed as previously described (Colt et al. 2004), except that ioxynil and 3,4-dichlorophenoxyacetic acid (3,4-D) were the extraction surrogates and the cleaned extracts were derivatized by methylation. Laboratory spikes (concentrations from 1,000 ng/g for most herbicides to 4,000 ng/g for bromoxynil and bentazon) of nine samples indicated that all target analytes were efficiently extracted with recovery means ranging from 71 to 124% and recovery standard deviations from 8 to 29%, except for fenoxyprop ethyl (149 ± 32%) and fluazifop-*p*-butyl (152 ± 26%). Reported levels in dust were not adjusted for spike recoveries. We conservatively estimated the detection limits as a concentration 0.1 ng/g below the lowest detected value, because many of these analytes had not been previously measured in dust. Detection limits were in the range of 24–62 ng/g dust except for dicamba (75 ng/g), 2,4-D (85 ng/g), bentazone (88 ng/g), pendimethalin (141 ng/g), and bromoxynil (231 ng/g) (Table 1). Improvements can be made by using labeled analytes as internal standards to compensate for matrix effects and for extraction inefficiency. However, because most of these standards are not commercially available and must be synthesized, the cost of such standards was beyond the budget for this project.

Eighty-eight percent of the dust samples (*n* = 112) were successfully analyzed for the target analytes. Reasons we did not analyze the others were that the identification label fell off the dust bag before lab receipt (*n* = 3), there was an insufficient quantity of sieved fine dust (*n* = 3), or laboratory error (*n* = 10). The laboratory error was the result of samples inadvertently being left at room temperature with some fluorescent light exposure for an extended period.

The frequency of detection of individual herbicides was often low, and interfering compounds that eluted together with the target analytes resulted in various types of “missing data.” Pesticides with > 20% of samples with interferences were pendimethalin (38% of samples), atrazine (29%), and fluazifop-*p*-butyl (21%). The distributions of the individual herbicide concentrations were consistent with log-normal distributions. We used a multiple imputation method, which assigns a value for each missing measurement by selecting a value from the assumed log-normal distribution based on a linear regression model (Lubin et al. 2004). Including factors significantly associated with herbicide concentration in dust (farming status, location of home inside or outside a town, acreage of crops within specific distances of home) in the imputation did not appreciably change our results, so we used imputed values from a regression model without covariates in our final analyses. Maximum likelihood parameter estimates were used to “fill in” five imputed concentrations for measurements that were below the detection limit or imprecisely reported because of interferences (Helsel 1990; Lubin et al. 2004; Moschandreas et al. 2001).

### Data analysis

Metribuzin and cyanazine were not detected in any samples. Bromoxynil and fenoxyprop ethyl were detected in < 5% of dust samples and were excluded from analyses. We grouped the remaining herbicides two ways for our analyses. First, we summed the concentrations of the six herbicides used almost exclusively in agriculture (acetochlor, alachlor, atrazine, bentazone, fluazifop-*p*-butyl, metolachlor; called here agricultural herbicides). Second, we evaluated the four detected herbicides that were used on ≥15% acres of corn and/or soybeans in Iowa in all three pesticide-use reporting years, 1985, 1990, and 1995 (atrazine, dicamba, metolachlor, trifluralin; called long-term–use herbicides). The herbicides accounting for the highest treated acreage of corn and soybeans, respectively, were atrazine (67% of corn acres treated in 1995) and trifluralin (30% of soybean acres treated in 1995). We also evaluated the concentration of individual herbicides that were detected in at least 5% of the samples (Table 1).

The descriptive statistics of the herbicide concentrations were based on the observed concentrations and the concentrations from one imputation, whereas the percent detections did not include any imputed values over the detection limit. We calculated percent detections and concentrations of the agricultural herbicide group separately for homes with and without an agricultural worker and by the location of residences within and outside of towns.

For each home, we determined the acreage of corn and soybeans within 750 m of the residence for each crop map year (1998–2000). We chose 750 m because primary pesticide drift from ground and aerial spraying—the most common methods of application of herbicides to corn and soybeans in Iowa—occurs within this distance (AgDRIFT Task Force 2002; Woods et al. 2001). We also determined the acres of corn and soybeans within “donut-shaped” buffer zones of < 100, 101–250, 251–500, and 501–750 m from homes. The acres of each crop type in the buffer zones changed little over the 3 years; therefore, we averaged the acreage across the 3 years. Because the average acreage of corn and soybean fields within each buffer zone was highly correlated [*r* ranged from 0.78 (100 m) to 0.90 (501–750 m)], we evaluated the summed acres of corn and soybeans in all analyses.

We conducted two main types of analyses. First, we used logistic regression to compute odds ratios (ORs) and 95% confidence intervals (CIs) of detecting one or more herbicides in each herbicide group in relation to the crop acreage anywhere within 750 m of the home and in relation to acres of crops within each buffer zone. Second, we used linear regression to model the logarithm of the concentration in relation to crop acreage anywhere within 750 m of the home and in relation to acres within each buffer zone. Linear regression models were run for each of the five data sets that contained the measured and imputed concentrations. Final parameter estimates and CIs were determined using the SAS procedure MIANALYZE (version 8.02; SAS Institute Inc., Cary, NC, USA). We computed likelihood ratio tests comparing the significance of nested models using models based on one imputed value. All analyses were evaluated for confounding by the presence of a current or past farmer in the home and whether the home was inside or outside a town. The ORs for herbicide detections were adjusted for agricultural employment because adjustment resulted in a change of 10% or more.

## Results

Detection rates for individual herbicides ranged from 0% for metribuzin and cyanazine to 95% for 2,4-D (Table 1). One or more of the six agricultural herbicides were detected in 28% of homes, whereas the four long-term–use herbicides were detected in 43% of homes. A few herbicides with high use (e.g., atrazine) were not detected frequently. Metolachlor was the most frequently detected herbicide in the agricultural herbicide group, whereas dicamba was the most frequently detected among the long-term–use herbicides. All homes with detections of one or more of the agricultural and long-term–use herbicides also had detections of 2,4-D. The concentration of 2,4-D was > 2-fold higher than any other herbicide. Herbicides with the highest geometric mean concentrations were 2,4-D, dicamba, pendimethalin, and acetochlor.

Homes where the respondent was currently employed in agricultural work had the highest frequency of detections of one or more of the six agricultural herbicides (85%), compared with homes of past farmers (37%) and homes of respondents who had never farmed or worked in jobs with pesticide exposure (16%) (Table 2). The geometric mean concentrations of agricultural herbicides in dust were about 3-fold higher among current agricultural workers’ homes compared with past workers and those never employed in these jobs.

Most residences (72%) were located in towns. Agricultural herbicide detections in residences in towns were less frequent (15%) than in rural residences (61%), and the herbicide concentration was about 2.5-fold higher in rural homes (Table 2). At increasing buffer zone distances, the percentage of homes with crops in the zone increased and was always greater for rural homes (data not shown). For example, the percentage of homes with crops within 500 m was 58% (town, 44%; rural, 94%). Likewise, the median acreage in each zone was substantially higher for rural residences; for example, at 501–750 m, the median acreage was 171 for rural homes compared with 20 acres for town residences. The maximum acres in each zone are constrained by the differing zone areas (range, < 100 m = 7.8 acres to 501–750 m = 243 acres). The frequency of detections and concentrations of agricultural herbicide increased with increasing acreage within 750 m (Table 2). Seventy-five percent of homes with > 300 acres of crops within 750 m had detections of agricultural herbicides. The concentrations of the agricultural herbicides were more than 4-fold higher compared with homes with no crops within 750 m.

Increasing acreage of corn and soybeans within 750 m of the home was associated with an increased probability of detecting one or more of the agricultural herbicides (Table 3). Compared with homes with no crops within 750 m, homes with ≥201 acres within 750 m were associated with significantly elevated ORs for detecting one or more of the agricultural herbicides. ORs were somewhat attenuated after adjustment for agricultural employment but were still significantly elevated. Each 10-acre increase in crops within 750 m was associated with a 6% increase in herbicide detections (adjusted OR = 1.06; 95% CI, 1.02–1.11). Increasing acreage within 750 m was also positively associated with the probability of detecting the long-term–use herbicides, although the estimate for the highest acreage category was unstable because only one home had no detection of one of the herbicides with long-term use (Table 3). For both the agricultural herbicides and long-term–use herbicides, decreasing distance of the crop acreage from the home (evaluated as acres within the four buffer regions) did not explain significantly more of the variance in herbicide detections compared with the models with crop acreage anywhere within 750 m (data not shown). The distance to the closest crop field was significantly associated with detections of the agricultural herbicide group; however, distance alone explained less of the variance than acreage within buffer zones of 100, 250, 500, and 750 m (data not shown).

The concentration of agricultural herbicides was significantly associated with the total crop acreage within 750 m of the homes (Table 4). Adjusting for presence of an agricultural worker in the home did not improve the model fit and did not change the parameter estimates substantially (data not shown). For each 10-acre increase in crops within 750 m, there was a significant increase (1.05-fold) in agricultural herbicide concentration. For example, among those with 200 acres of crops near their home, there was a 2.7-fold (1.05^20^) increase in herbicide concentration compared with those with no crops within 750 m. Including separate parameters for crop acres in each buffer zone did not significantly improve the model fit (*p* > 0.05), and there was no clear pattern in the relationship between herbicide concentrations and distance of acres from the home (Table 4). However, each acre within the 100–250 m buffer region was associated with a marginally significant increase in herbicide concentrations (e.g., 2.4-fold increase for 10 acres). For the group of four herbicides with high use in all time periods, we observed a similar and somewhat stronger relationship between crop acres within 750 m and herbicide concentrations (Table 4). For each 10-acre increase in crops within 750 m, there was a significant 1.06-fold increase in herbicide concentrations. Including terms for crop acreage within the four buffer zones did not improve the model fit significantly (*p* > 0.05).

We evaluated the association between the concentration of individual herbicides detected in > 5% of homes and both crop acreage within 750 m and crop acreage within each buffer region. Total acreage within 750 m was significantly (*p* < 0.05) associated with the concentration of 2,4-D, dicamba, and metolachlor but not with atrazine, acetochlor, alachlor, pendimethalin, trifluralin, fluazifop-*p*-butyl, or bentazone (data not shown). Including terms for the distance of the acreage from homes did not significantly improve the model fit for any of these herbicides.

## Discussion

In the U.S. Midwest, > 90% of corn and soybean fields are treated with herbicides. We found that increasing acreage of corn and soybean fields within 750 m of homes was associated with a greater probability of detecting one or more of the major corn and soybean herbicides in homes. The total concentrations of agricultural and long-term–use herbicides were significantly associated with increasing acreage within 750 m of homes; likewise, concentrations of the herbicides 2,4-D, dicamba, and metolachlor were also associated with increasing acreage. Employment in agriculture was positively associated with the presence and concentrations of the herbicides in homes; however, the relationship between agricultural herbicides detections and levels in dust and crop acreage remained after adjusting for agricultural employment.

Previous studies in Washington State found a positive relationship between the self-reported proximity of homes to orchards and both the concentrations of organophosphate insecticides in house dust and levels of the metabolites in children’s urine (Fenske et al. 2002; Simcox et al. 1995). In those studies, levels increased with increasing proximity of homes to crops over distances ranging from approximately 15 to 400 m. Curwin et al. (2005) compared dust concentrations of several of the same corn and soybean herbicides that we measured (atrazine, acetochlor, metolachlor, 2,4-D) in farm and nonfarm homes in Iowa and found that self-reported distance to treated fields was not associated with herbicide levels in dust among nonfarm homes. However, the closest distance category was < 0.25 mile (402 m). Curwin et al. (2005) did not evaluate distance to crop fields among farm homes because all were reported to be < 402 m from a treated field. Both studies relied on self-reported estimates of distances to crop fields, which may introduce misclassification. Neither study mapped the crops near homes, which would have allowed for an objective evaluation of both proximity and acreage of crops near homes.

We evaluated the relationship of acreage within specific distance intervals (donut-shaped circular buffers) with herbicide concentrations. We found that the distance of the crop acreage from homes did not explain significantly more of the variance in herbicide detections and concentrations compared with the total crop acreage within a 750-m buffer, nor was there a clear pattern of increase in herbicide concentrations with proximity. The crop acreage in adjacent buffer zones was highly correlated (*r* > 0.88). Because of the large size of crop fields in Iowa, all homes with crops within 100 m and 101–250 m also had crop acreage at farther distances.

Because we evaluated acreage of crops within specific distance intervals rather than the distance to the nearest crop field, our analyses cannot be directly compared with those of Simcox et al. (1995) and Curwin et al. (2005). Those studies evaluated only the relationship between distance of crop fields from homes and pesticide levels in house dust and did not account for the size of crop fields near homes. A metric based on distance alone assumes that a large crop field and a small crop field at the same distance have the same influence on residential herbicide levels. We determined that increasing acreage within 750 m of homes was a significant predictor of herbicide levels in dust. However, acreage was highly correlated with increasing distance in our study, and the resultant collinearity could have resulted in the lack of a clear relationship between the proximity of crop acreage and herbicide concentrations. A high degree of collinearity between predictive variables can result in poor parameter estimations with high variances (Kleinbaum et al. 1998).

Our analysis of the relationship between herbicide concentrations and crop acres near the home was limited to acreage within 750 m. Therefore, additional studies to evaluate acreage beyond 750 m will be important. Development of a metric that addresses weaknesses inherent in the methods used to date (e.g., weighted measurements of acreage) is needed to evaluate the relationship between the acreage of crop fields in proximity to homes and residential pesticide levels. Further research is also needed to determine if other factors such as meteorologic conditions, pesticide transport associated with wind-blown aerosols and soil (secondary drift), and physicochemical properties of herbicides are important predictors of herbicide concentrations in environmental and biologic samples. For example, the vapor pressure of the pesticide and meteorologic conditions are important factors in the estimation of exposure to drift from agricultural pesticide applications (Lee et al. 2002; Ramaprasad et al. 2004). These factors and local variation in herbicide use are likely to explain why the detections of herbicides in dust samples did not directly correspond with agricultural herbicide use rates.

Previous studies have demonstrated the importance of the “take-home” pathway of exposure for families living with an agricultural worker (Curl et al. 2002; Lu et al. 2000), which we confirmed. We found that currently active agricultural workers had substantially higher frequencies of detection of agricultural herbicides in their homes than did former agricultural workers and nonfarmers. Concentrations of these pesticides were > 4-fold higher in homes of current agricultural workers compared with homes with no agricultural workers. A total of 72% of our study population resided within town boundaries, compared with 91% in a similar analysis in Nebraska by Ward et al. (2000). Among residences within towns, 74% had crops within 750 m of the home, and 15% of homes in town had detections of one or more agricultural herbicide; thus, residence in a town may not preclude exposure to crop pesticides.

Proximity to pesticide applications as reported in the California Pesticide Use Reporting (CPUR) database has been used as a surrogate for exposure in recent studies of reproductive outcomes and cancer (Bell et al. 2001; Reynolds et al. 2002), but the relationship between proximity and human exposure in those studies was not determined. A recent study using the CPUR data demonstrates that estimates of residential proximity to pesticide applications can differ substantially depending on the distance used to calculate the metric (Rull and Ritz 2003), with potentially large effects on risk estimates. Our study indicates that residential exposure to commonly used agricultural herbicides is higher among those living within 750 m of agricultural fields and among those with an agricultural worker in the home. Further research is needed to determine how well measurements of corn and soybean herbicides in residential dust samples predict human exposure. Results of this study suggest that satellite-based crop maps may be a useful method for estimating levels of herbicides in homes from nearby crop fields and thereby serve as a surrogate measure of potential exposure to agricultural pesticides.

## Figures and Tables

**Table 1:** Frequency of detection, median detected concentrations (ng/g), geometric mean, and geometric SD for 14 herbicides measured in house dust samples from Iowa residences.

Herbicide	Percent detected^a^ (*n* = 112)	Median detected concentration (range)	Geometric mean^b^	Geometric SD
2,4-D^c^	94.6	1244.9 (85.2–126,000)	1035.0	4.2
Dicamba^c^	28.8	179.5 (75.4–908.0)	30.1	5.0
Metolachlor	21.4	129.5 (27.8–3180.0)	3.0	16.8
Trifluralin	15.2	51.9 (24.6–3650.0)	1.5	17.0
Pendimethalin	11.6	215.0 (141–1650.0)	17.5	6.1
Atrazine	8.0	510.0 (59.4–4720.0)	0.44	41.4
Bentazon^c^	8.1	225.0 (88.5–855.6)	5.6	6.4
Acetochlor	8.0	127.0 (52.7–4120.0)	16.5	3.1
Fluazifop-*p*-butyl	6.2	71.3 (29.1–304.0)	2.3	5.9
Alachlor	6.2	49.6 (28.4–394.0)	3.2	4.3
All herbicides^c^	94.6	1444.2 (173.3–126,190)	1398.6	3.5
Agricultural herbicides^c^,^d^	28.0	238.3 (34.7–10244.0)	72.5	3.5
Herbicides with long-term use^e^	43.2	193.5 (31.6–6469.0)	74.7	4.5

a Detection limits (ng/g): acetochlor, 53; alachlor, 28; atrazine, 59; bentazon, 88; dicamba, 75; fluazifop-*p*-butyl, 29; metolachlor, 28; pendimethalin, 141; trifluralin, 24; 2,4-D, 85.

b Geometric means and SDs were derived from fitted log-normal models.

c Based on 111 samples.

d Herbicides with primarily agricultural use: acetochlor, alachor, atrazine, bentazon, fluazifop-*p*-butyl, metolachlor.

e Herbicides used on ≥15% of corn and/or soybean acreage in Iowa in 1985, 1990, and 1995: atrazine, dicamba, metolachlor, trifluralin.

**Table 2:** Frequency of detections, geometric mean, and geometric SD for agricultural herbicides measured in house dust, by agricultural work status, residence location, and acres of crops near the homes.

	No. (%)	Percent of homes with detections	Geometric mean (ng/g) (geometric SD)
Agricultural work			
Never	79 (71)	16	111.5 (2.5)
Current	13 (12)	85	366.0 (4.6)
Former	19 (17)	37	121.9 (2.5)
Residence in town			
Yes	80 (72)	15	101.7 (2.2)
No	31 (28)	61	245.7 (4.2)
Acres of corn and soybeans within 750 m of home			
0	29 (26)	14	45.2 (2.0)
1–200	58 (52)	17	63.1 (3.2)
201–300	8 (7)	62	143.3 (2.4)
≥301	16 (14)	75	200.3 (6.3)

**Table 3:** ORs and 95% CIs of detecting one or more herbicides in relation to the total acreage of corn and soybeans within 750 m of residences.

	Acres of corn and soybeans within 750 m^a^								
	No crops within 750 m^b^		1–200 acres		201–300 acres		> 300 acres		
Herbicide	Detect/ND^c^	OR	Detect/ND^c^	OR (95% CI)	Detect/ND^c^	OR (95% CI)	Detect/ND^c^	OR (95% CI)	OR (95% CI) per 10 acres
Agricultural herbicides									
Crude OR	5/24	1.0	10/48	1.3 (0.4–4.5)	5/3	10.4 (1.8–61.7)	12/4	18.7 (3.9–88.1)	1.09 (1.05–1.13)
Adjusted for agricultural jobs		1.0		1.2 (0.3–4.4)		7.2 (1.1–49.3)		7.4 (1.3–41.3)	1.06 (1.02–1.11)
Long-term–use herbicides									
Crude OR	7/22	1.0	21/37	1.8 (0.7–4.9)	5/3	5.2 (1.0–27.7)	15/1	47.1 (5.2–423)	1.08 (1.04–1.12)
Adjusted for agricultural jobs		1.0		1.7 (0.6–5.1)		3.6 (0.6–22.9)		19.9 (2.0–201)	1.05 (1.01–1.10)

a Average acreage in 1998–2000.

b Reference group.

c Number of homes where herbicides were detected/number of homes where herbicides were not detected (ND).

**Table 4:** Exponentiated parameter estimates for agricultural herbicides and long-term–use herbicides in carpet dust (ng/g) in relation to acres of crops anywhere within 750 m and within specific buffer regions from home (*n* = 111 samples for each model).

Model	Parameter	Exp(β)	95% CI
Agricultural herbicides			
Model A	Intercept	64.02	47.31–86.63
Model B	Intercept	38.44	24.88–59.37
	Per 10 acres: < 750 m	1.05	1.03–1.07
Model C	Intercept	40.90	24.85–67.32
	Per acre: < 100 m	0.99	0.58–1.68
	Per acre: 100–250 m	1.09	0.98–1.21
	Per acre: 250–500 m	0.98	0.95–1.01
	Per acre: 500–750 m	1.01	1.00–1.02
Long-term–use herbicides			
Model A	Intercept	76.90	55.85–105.87
Model B	Intercept	36.86	24.70–55.01
	Per 10 acres: < 750 m	1.06	1.04–1.08
Model C	Intercept	39.66	25.94–60.62
	Per acre: < 100 m	0.77	0.43–1.40
	Per acre: 100–250 m	1.12	1.00–1.27
	Per acre: 250–500 m	0.98	0.95–1.02
	Per acre: 500–750 m	1.01	1.00–1.02
